# Carbon and Graphene Coatings for the Thermal Management of Sustainable LMP Batteries for Automotive Applications

**DOI:** 10.3390/ma15217744

**Published:** 2022-11-03

**Authors:** Luigi Sequino, Gaetano Sebastianelli, Bianca Maria Vaglieco

**Affiliations:** Istituto di Scienze e Tecnologie per l’Energia e la Mobilità Sostenibili—CNR, 80125 Napoli, Italy

**Keywords:** carbon coating, graphene coating, Li-ion batteries

## Abstract

The increment of battery temperature during the operation caused by internal heat generation is one of the main issues to face in the management of storage systems for automotive and power generation applications. The temperature strongly affects the battery efficiency, granting the best performance in a limited range. The investigation and testing of materials for the improvement of heat dissipation are crucial for modern battery systems that must provide high power and energy density. This study presents an analysis of the thermal behavior of a lithium-polymer cell, which can be stacked in a battery pack for electric vehicles. The cell is sheltered with layers of two different materials: carbon and graphene, used in turn, to dissipate the heat generated during the operation in natural convection. Optical diagnostics in the infrared band is used to evaluate the battery surface temperature and the effect of the coatings. Experiments are performed in two operating conditions varying the current demand. Moreover, two theoretical correlations are used to estimate the thermal parameters of the battery with a reverse-logic approach. The convective heat transfer coefficient h and the specific heat capacity c_p_ of the battery are evaluated and provided for the Li-ion battery under investigation for different coatings’ conductivity. The results highlight the advantage of using a coating and the effect of the coating properties to reduce the battery temperature under operation. In particular, graphene is preferable because it provides the lowest battery temperature in the most intense operating condition.

## 1. Introduction

Future sustainable mobility identifies hybrid electric vehicles as the core of the next urban transportation system [1,2,3,4]. To address the challenges of this transition [5,6,7,8] it is necessary to control one of the main problems of batteries which is thermal management. The management of the thermal behavior of the battery is crucial to the safety, efficiency, and life of the battery pack, in both hybrid and fully electric vehicles. The heating of the battery is a well-known aspect [6,9,10] and is linked to its operation; the greater the load, the greater the heat generation [11,12]. Uneven temperature distribution and its local peaks are critical during charge and discharge cycles. Batteries are exposed to the risk of thermal runaway, a catastrophic event triggered by local heat concentrations, that has greatly raised public concerns [13,14]. Thermal management issues [8] have been a limitation to the diffusion of lithium-ion batteries (LIB) in electric vehicles (EVs) and for this reason there is an urgent need to design safer batteries. Internal short circuit (ISC) is frequent among battery failures [15]. ISC may trigger or accelerate thermal runaway (TR) by contributing to the temperature increment and the start of a series of exothermic side reactions. To manage the thermal status of the battery pack, a cooling system can be applied. Different configurations are widely tested according to the operating conditions, available volume, and battery pack performance. Chen et al. [16] carried out a numerical investigation to compare different cooling methods for lithium battery cells, obtaining interesting insights to consider for the cooling system selection and design. For example, the system with fins was seen to add the most extra weight; whereas the air cooling consumed the most parasitic power, and the indirect liquid cooling system had the highest maximum temperature difference. Numerical investigations are very helpful for the analysis of dangerous and unstable processes such as thermal runaway. To improve the management of thermal behavior and to run numerical simulations, it is essential to identify the main thermal properties of the batteries, such as specific thermal capacity, thermal conductivity, and convective heat transfer coefficient. Zhang et al. presented a way to simultaneously estimate these parameters using the inverse heat transfer formulation [17]. In place of directly calculating them, a first simulation was run with initial values, then they made a comparison between calculated and measured temperatures, and the following refinement of the values of the parameters was made until the optimized set of values was found. Sheng et al. [18] developed a novel method to determine the thermal conductivity and the specific heat simultaneously based on the quasi-steady state heat transfer analysis. They found that the operating temperature has a more significant influence on the cell-specific heat than the thermal conductivity, while the effect of the state of charge has a minimal effect on these two parameters. The importance of a reliable determination of the thermal parameters is crucial, as highlighted by Dong et al. [19] who evaluated the thermal model inaccuracy caused by physical properties uncertainty. While providing reliable values of battery temperature both internal and external, the convective heat transfer coefficient can be relevant for the prediction and prevention of over-temperature risk. To improve the rate of heat exchange from the battery to the cooling means, independently of the heat transfer method used, high thermal properties materials can be used to coat the battery. In this regard, some experimental applications can be found with the use of carbon- and graphene-based materials [20,21,22,23] but only as coatings for the anode and the cathode. Carbon can be used to improve the performance of the electrodes, as shown by Wee et al. [20], who carried out a systematic study on the microstructural effect of carbon materials on the electrochemical or electrocatalytic performance of the electrodes. Moreover, Park et al. [21] investigated the effect of carbon coatings on the thermal stability of natural graphite spheres used as anode materials in lithium-ion batteries by applying differential scanning calorimetry and X-ray diffraction. Among carbon-based materials, graphene is of particular interest because of its outstanding mechanical flexibility and ultralow weight, which can adapt to different applications that can grant structural support and thermal dissipation properties at the same time. Graphene has an impressive capability of electrolyte infiltration, which could accelerate the Li-ion’s diffusion [22] providing a solution for key issues affecting the cathode of future lithium-sulfur batteries. Graphene-based hybrid electrodes have been exploited to increase electron transport, specific capacity, C-rate, and cyclability. Graphene can be used as a substrate for the growth of anode/cathode nanomaterials to achieve higher rate performance electrodes compared to non-conducting materials [24]. There are several methods for the synthesis of graphene, from electrochemical exfoliation through rapid thermal annealing to biomass pyrolysis, each of them with its benefits and challenges [25]. Moreover, the electrochemical properties of graphene and carbon nanotubes can be tailored by introducing heteroatoms into the carbon crystalline lattice granting to improve the kinetics of the heterogeneous electron transfer reaction [26]. Besides these typical applications of coatings inside the battery linked to the electrochemical aspects of carbon and graphene, there are also some uses outside as matrix structures for heat dissipation with phase change materials (PCM). Researchers have developed a variety of methods to increase the thermal conductivity of PCM by adding high thermal conductivity materials [27] (e.g., adding carbon fiber, metal foam, expanded graphite [28], or adding fin structure). They show that increasing the thermal conductivity of PCM can effectively improve the heat dissipation performance of BTMS. While poor, literature can be found about covering the battery surface with high-conductivity coatings. Saw et al. [29] proposed a feasibility study of boron nitride on Li-ion battery casing to enhance thermal management, also paying attention to the effect of the surface roughness and the coating thickness on the heat transfer. However, there is no evidence in the literature of the use of carbon fiber-reinforced polymer (CFRP) composites that are largely widespread thanks to their excellent mechanical properties as low density, high specific strength, and modulus [30,31,32]. Hence, from the thermal point of view, there is room for a wide experimental investigation on carbon and graphene-based surface coatings for heat dissipation to preserve battery health and ensure a safe operation.

This work presents an experimental analysis of the thermal state of a flat-type battery coated with layers of carbon and graphene. The battery surface temperature is detected using infrared thermography during discharge processes at a constant current and a charging phase. Thermography has proven to be a reliable and practical tool for studying the thermal behavior of LIBs by performing non-invasive measurements of the surface temperature over time. This work presents and discusses the efficacy of two methods for the fast evaluation of the convective heat transfer coefficient h, and the specific heat capacity c_p_ of the battery. The first is a 1d model based on the finite difference method, which can characterize the thermal status inside and on the surface of the battery. The second method uses Newton’s law of cooling for the direct calculation of the thermal parameters. The outputs of the two methods are compared using the normalized root mean squared error producing a positive agreement. This work provides numerical values of the main thermal parameters of a common Li-ion battery under different load conditions. By addressing the effect of the coating conductivity, it offers a comparative analysis that can be transferred to different types of cells and geometries for a preliminary analysis. Finally, also the proposed methodology is presented to be re-applied to different cases and battery configurations.

## 2. Experimental Apparatus

### 2.1. Battery

**Figure 1:**
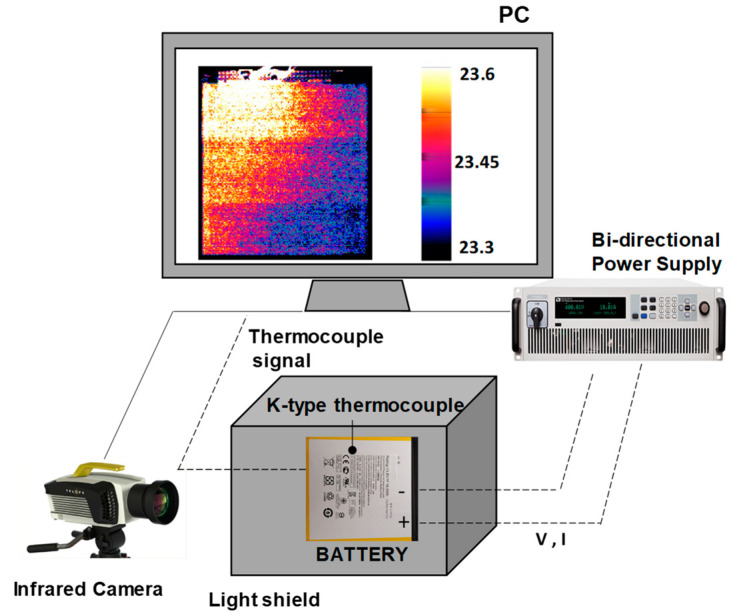
Scheme of the experimental setup. The infrared camera, the battery in the test box, the bi-directional power supply, and a sample image of the battery in the infrared band are reported.

Figure 1 reports the experimental setup used in this study. The battery is a lithium metal polymer battery (LMP) with a capacity of 3300 mAh, a discharge nominal voltage of 3.8 V, and a rated power of 18.5 Wh. The battery dimensions are 100 × 110 × 3 mm^3^, the heat exchange area is 22,000 mm^2^ and the mass is 75 g, giving a specific capacity of 65.2 mAh/g. The positive current collector is made of aluminum and the negative of copper. The cell features a LiMn_2_O_4_ cathode and a LiC_6_ (graphite) anode. 2D imaging is used for thermal characterization due to the high sensitivity and spatial resolution compared to thermocouples for the cases under investigation. Low current values produce indeed small temperature variations. This preliminary investigation of surface temperature homogeneity is mandatory for battery modeling. The experiments are run using a box realized on purpose. To avoid the incidence of infrared radiation from the surrounding environment on the battery, the inner walls are painted black. The battery is placed on adiabatic supports to avoid undesired heat losses. The face of the battery inspected by the camera is covered with a material with a high emissivity to avoid reflection from the surrounding ambient and to increase the accuracy of the measurements. The environmental conditions are controlled at dry-bulb temperature and relative humidity.

### 2.2. Coatings

**Figure 2:**
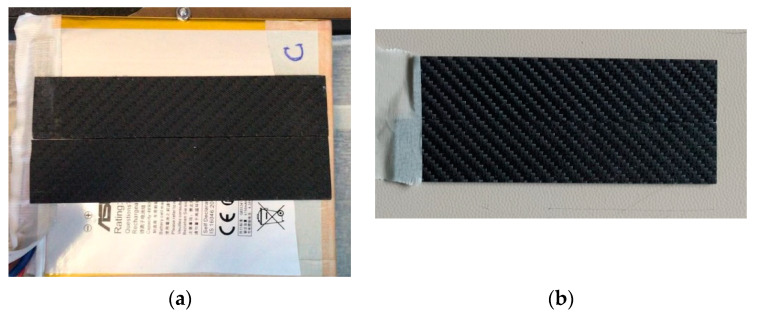
Image of the battery with the carbon layer (**a**) and of the graphene layer before the assembling (**b**).

To dissipate the heat generated by the battery, coatings of different materials are applied. The study is first conducted on an uncoated battery that serves as a reference and subsequently uses coatings of different materials. The first is a carbon fiber-reinforced polymer (CFRP) composite while for the second one a further coating of graphene nano-platelets is applied to the carbon-fiber polymer. The CFRP is a laminate sheet produced with a 57% fiber volume fraction. The laminate comprised four plies of woven bi-directional carbon fiber fabric bonded with bisphenol-based epoxy resin in combination with a hardener, according to the procedure reported in [32]. For this study, the laminate thickness is 1.5 mm. In the following, the terms carbon and graphene layers will be used to refer to the CFRP composite materials. As prototype components, their thermal characteristics are unknown and then tests are performed on purpose to evaluate the thermal conductivity. Also, the battery conductivity across the transverse profile is evaluated with the same procedure. The guarded hot plate method is applied, it is a steady-state method that involves measuring the thermal gradient by applying a known heat flow to one side of the body. As highlighted in a recent work by White et al. [33], the ideal 1D heat flow is in reality multi-dimensional because a favorable heat path around the cell case exists which causes an overestimation of the thermal conductivity. For example, Steinhardt et al. estimated an error of about +20% on a 34 Ah prismatic cell when subtracting the heat flow around the cell sides [34]. In this work, a battery conductivity of 1.25 W/mK is calculated across 3 mm thickness which is typically much lower than a prismatic cell as used in the previous literature study [34]. Scaling 1.25 W/mK by 20% would still give 1.03 W/mK which is in line with the range 0.3–1.3 W/mK found in the literature [35,36]. For the coatings, the estimated mean thermal conductivities are 0.67 and 0.86 W/mK for the carbon and graphene, respectively, as reported in Table 1. The conductivity calculated for the graphene also agrees with the values found by Zou et al. [37] where graphene and carbon nanotubes were used as additives for PCM. The coatings are applied to the rear side of the battery, opposite the camera using a thin layer of silicone thermal grease with a thermal conductivity close to that of the battery (1.4 W/mK). Figure 2 shows photographs of the battery covered with the carbon layer (Figure 2a) and of the graphene layer before the assembly (Figure 2b).

### 2.3. Measurements Procedures

The battery evaluations consist of two discharge and one charge test, the resting phase that follows the discharge is also analyzed. The discharge is run until a SOC of 50%. The current rate of 1C (3.3 A or a current density of 0.044 A/g)) which would completely discharge the battery in 1 h, is applied for 0.5 h; while, for the value of 0.5C (1.65 A or a current density of 0.022 A/g), the test duration is 1 h. The resting phase is recorded for about 15 min to make the battery temperature return to the ambient temperature, the test specifications are defined in Table 2. The voltage and the current signals are examined using a bi-directional power supply. An infrared detector in front of the battery records thermal radiation by the inspection access of the testing box. The infrared camera reveals the 2D temperature distribution; it has a resolution of 320 × 256 pixels and is sensitive in the range of 3.0–5.5 µm. The sensor is made of indium antimonide and a 50 mm lens is used (range 3–5 µm). The acquisition frequency is 0.1 Hz and the exposure time of 260 µs is set to get the best compromise between recording quality and image saturation. The camera is at 1 m from the battery. The thermal camera calibration is executed and regularly checked by the supplier. Also, a K-type thermocouple is installed on the battery surface to monitor the local temperature. The thermocouple is located in the top-left corner of the battery and has a sensitivity of 0.5 °C. The signal is recorded by an I/O analog module and a homemade acquisition interface.

### 2.4. Image Elaboration for Temperature Detection

The temperature value for each pixel is obtained by image processes. The temperature of the battery surface and the background are detected; the latter is indicative of the ambient temperature that is double-checked with a sensor. The raw pixel values are compensated by setting the emissivity of the material and the ambient temperature. The 2D temperature is evaluated to point out local peaks or temperature gradients. The images show a homogeneous 2D temperature distribution as visible in the high-contrast image of Figure 1. A maximum difference of temperature of 0.3 °C from the top left corner of the battery (current collectors) to the bottom right one can be identified. This relates to 1.3% of the average battery temperature; hence, a constant temperature can be considered over the surface, and then the lumped parameter model can be applied for the analysis of the thermal status. Inside each region of interest (ROI), the average temperature is evaluated by summing up the values of all the pixels and then dividing it by the total number of pixels in that region. This calculus is made both for the background and for the battery. Great attention is paid to avoid the edge effects. The average temperature for all the recorded images is plotted as a curve over time.

## 3. 1D thermal Modeling of the Battery

### 3.1. Finite-Difference Method

The battery heats up during functioning because of two main phenomena, the chemical reactions between the electrodes and the Joule effect generated by the current flow [8]. The power generated per surface unit, ${\dot{q}}_{gen}$, depends on the current and the voltage values according to the following equation:

(1) $${\dot{q}}_{gen}=-I\left[-\frac{dV_{oc}}{dT}T+\left(V_{oc}-V\right)\right]\cdot \frac{1}{A}$$

where *I* is the current, *V_oc_* is the open-circuit voltage, and *V* is the actual voltage. $\left(V_{oc}-V\right)I\;$ is the heat produced because of cell polarization and the term $-dV_{oc}/dT$ is due to the reversible entropy change in the cell. With the derivative term being very small (lower than 0.001 V/°C [38]), the main driver of the power loss is the difference between the open circuit and the actual voltage. The measure of the actual voltage can be performed online during the battery functioning, while the *V_oc_* can be measured only after a long rest time.

**Figure 3:**
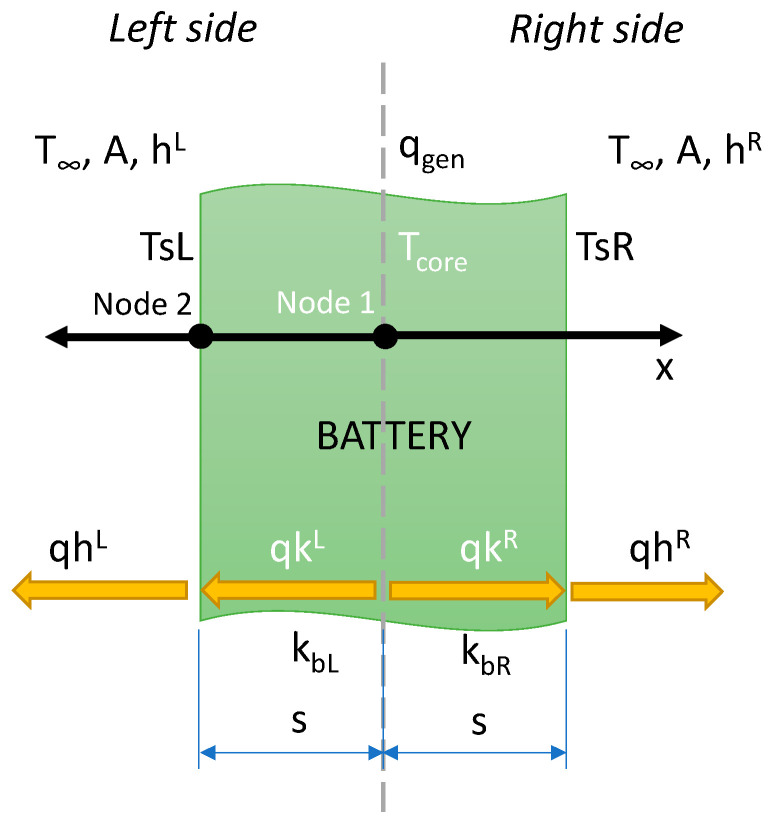
Scheme of the 1D model and air/battery thermal properties. The heat flux directions, the nodes labels, and the symmetry axis are reported.

The thermal status of the battery is described by the following balance:

(2) $$mc_{p}\frac{dT_{s}}{dt}={\dot{q}}_{gen}-hA\left(T_{s}-T_{\infty }\right)$$

where ‘*m*’ is the mass of the battery, ‘*c_p_*’, its specific heat capacity, ‘*t*’, the time, ‘*T_s_*’, the battery surface temperature, ‘*T*_∞_’, the ambient temperature, ‘*A*’, the battery surface area, and ‘*h*’, the convective heat transfer coefficient. The temperature of the battery increases because of the internal heat generated and decreases thanks to the heat exchange with the surrounding ambient. Figure 3 reports the scheme of the thermal model of the battery. The finite-difference method is applied to solve the heat transfer problem. The battery symmetry can be considered for the analysis considering only two nodes: the temperature in the core, ‘*T_c_*’, and that on the surface, ‘*T_s_*’. The equations used are:

Node 1 (core temperature)

(3) $$T_{c}\left(t+1\right)=T_{c}\left(t\right)+\left[{\dot{q}}_{gen}\left(t\right)-\frac{Ak_{bL}}{sL}\left(T_{c}\left(t\right)-T_{sL}\left(t\right)\right)-\frac{Ak_{bR}}{sR}\left(T_{c}\left(t\right)-T_{sR}\left(t\right)\right)\right]\cdot \frac{\Delta t}{mc_{p}}\;\;$$

where the suffixes ‘*L*’ and ‘*R*’ are used for the left and right sides, respectively; which are the same for the uncoated battery.

Node 2 (surface temperature)

(4) $$T_{sL}\left(t+1\right)=T_{sR}\left(t+1\right)=\left(hT_{\infty }+\frac{k_{b}}{s}T_{c}\left(t+1\right)\right)/\left(h+\frac{k_{b}}{s}\right)$$

where ‘*T_c_*’ and ‘*T_s_*’ are the unknown variable for each time step, ‘*k_b_*’ is the thermal conductivity of the battery, and ‘*s*’, is the thickness. For the case with no coating, *T_s_* is the same on both sides of the battery. When the coating is applied, the thickness and conductivity are different for the left and the right sides of the battery. Equation (4) is doubled for *T_sL_* and *T_sR_*, for the left side no modifications are needed; for the right side with coating, there are the total thickness (*s_R_ = s*_coating_ + *s*), and the equivalent conductivity:

(5) $$k_{eq}=\frac{s_{c}+s}{\frac{s_{c}}{k_{c}}+\frac{s}{k_{b}}}$$

where *k_c_* and *s_c_* are the conductivity and the thickness of the coating, respectively.

### 3.2. Evaluation of the Thermal Parameters

Equations (3) and (4) are used to evaluate the internal and surface temperature of the battery given some geometrical and thermal parameters. While the battery geometry and mass are known, the conductivity of the materials, the specific heat capacity, and the convective heat transfer coefficient must be evaluated. The conductivity of the battery and the coatings are experimentally measured in the laboratory and have been previously illustrated. The convective heat transfer coefficient cannot be measured directly; moreover, it depends on the working conditions. Some correlations are reported in the literature [39] considering the body shape and the non-dimensional numbers as Rayleigh and Prandtl, for natural convection. Similarly, the specific heat capacity of the battery cannot be easily calculated due to its non-isotropic characteristics, multiple layers of anode–electrolyte–cathode alternate inside the battery casing. Also, for the coatings, the specific heat capacity is unknown. To overcome this issue, the curves of temperature are simulated using a series of parameter values inside a certain range and then they are compared to the measured data. The Normalized Root Mean Squared Error (NRMSE) between experimental and model data is calculated to quantify the differences. The couple of *h* and *c_p_* that minimizes the NRMSE is selected. This procedure provides the convective heat transfer coefficient on the free side of the battery and the equivalent specific heat capacity of the group battery + coating. This evaluation is performed only for the discharge tests at 1C and 0.5C.

### 3.3. Newton’s Law of Cooling

For the case under investigation, a simplification can be made by considering the lumped parameter model. The Biot Number, linked to the combined effects of convection and conduction, is much lower than 1 (Bi << 1) because of the small thickness of the battery [40]. Equation (1) allows evaluation of the theoretical heat generated ${\dot{q}}_{gen}$ and Newton’s law for transient conditions can describe the battery temperature behavior over time during the cooling and heating phases according to an exponential behavior.

(6) $$T=T_{\infty }+\left(T_{i}-T_{\infty }\right)e^{-\frac{hA}{mc_{p}}t}+\left[1-e^{-\frac{hA}{mc_{p}}t}\right]\cdot \frac{{\dot{q}}_{gen}}{h}$$

where T is the battery surface temperature, $T_{\infty }$ is the surrounding ambient temperature, $T_{i}$ is the initial temperature of the battery (that can be equal to the ambient temperature for the heating process or higher for the cooling process), h is the convective heat transfer coefficient, A is the battery area, m is the battery mass, and $c_{p}$ is the specific heat capacity. It can be observed that, unlike the finite-difference model, here the parameters relative to the internal characteristics of the battery such as conductivity and width are not considered. This strongly simplifies the elaboration, in particular when these data are not available. In the case of cooling, ${\dot{q}}_{gen}=0$ and Equation (6) simplifies as follows:

(7) $$T=T_{\infty }+\left(T_{i}-T_{\infty }\right)e^{-\frac{hA}{mc_{p}}t}$$

Conversely, during the discharge phase, ${\dot{q}}_{gen}\neq 0$ and the initial temperature $T_{i}$ is equal to the ambient temperature $T_{\infty }$; therefore, it becomes:

(8) $$T=T_{\infty }+\left[1-e^{-\frac{hA}{mc_{p}}t}\right]\cdot \frac{{\dot{q}}_{gen}}{h}$$

Equations (7) and (8) describe the evolution of the battery temperature during the heating and the cooling phases which are the discharge and the resting phases, respectively. The main thermal parameters affecting the temperature variation are h and $c_{p}$. These are strictly linked to the battery material and surrounding fluid properties; hence, it is fundamental to evaluate them for accurate temperature estimation. Equations (7) and (8) are then solved for *h* and $c_{p}$ for each time step. They vary over time, taking an overshoot at the start of the heating and cooling phases and then settling around a constant value taken as the average value.

### 3.4. Evaluation of the Reversible Entropy Parameter

**Figure 4:**
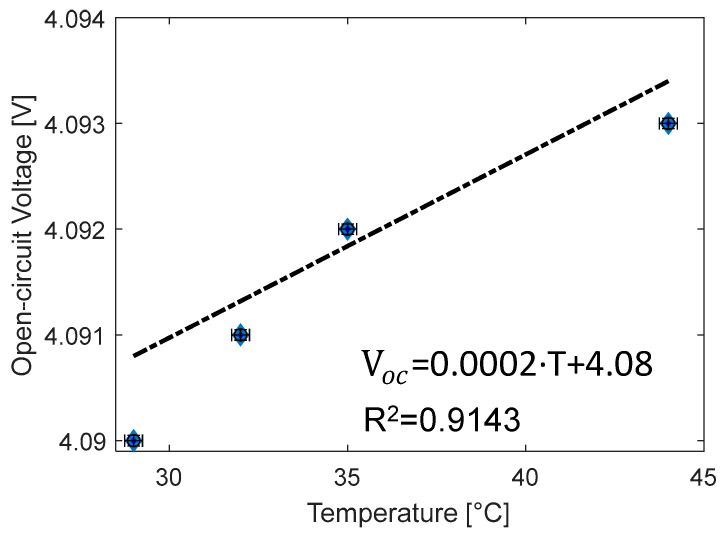
Determination of the coefficient of entropy of the battery calculated as the slope of the linear regression obtained from the experimental data.

The heat generated ${\dot{q}}_{gen}$ was calculated using Equation (1). The term $dV_{oc}/dT$ is related to the reversible heat generation due to the entropy variation; it is evaluated as the variation of the open-circuit voltage with the temperature. A value of 0.002 has been found in the literature [38], but the case under investigation does not produce a good agreement between the model and experimental values. Since the capability to model a thermal system of the battery is directly related to the accuracy of the determination of its reversible heat generation coefficient [41], for the tested battery, an evaluation has been performed on purpose. The experiment has been set in the laboratory, consisting in heating the battery inside a chamber and measuring the voltage using the bidirectional power supply. The variation of the open-circuit voltage is evaluated during the cooling phase to take advantage of a smoother and slower variation, the battery surface and the chamber are instrumented with thermocouples to measure the temperature. A constant SOC of 95% is selected to avoid heating up the fully-charged battery. Cooling down from 45 to 25 °C, the data are collected each 0.001 V and are reported in Figure 4. A linear regression of the measurements is performed to linearize the temperature dependence of the open-circuit voltage. The straight line passes through the cloud of points without crossing the error bars because a very low standard deviation is calculated (the maximum values is 7.1 × 10^−5^ V for the data at 25 °C). However, the coefficient of determination (R^2^) of the linear regression, reported in the figure, is satisfying. With a value of 0.9143, it indicates that the linear correlation is a good fit for the data. Finally, the dependence of the open-circuit voltage to the temperature, approximated with the term $dV_{oc}/dT$, is 0.0002 V/°C. This is one order of magnitude lower than the values from the literature and, moreover, it is in line with some recent studies. In fact, for Lenz et al. [42] with an NMC battery, it ranges between 0.00005 and 0.0003 V/°C in a non-linear way increasing the SOC, while Wang et al. [43], for a pouch lithium-ion cell, have 0.00022 V/°C.

## 4. Results and Discussion

In this section, the effect of coatings of different materials and conductivities on the thermal status of the battery is investigated via thermal imaging and theoretical models. First, the electric and thermal parameters are experimentally investigated to understand the battery behavior and the processes that regulate its thermal status. Then, a model-based analysis is performed to estimate the battery thermal parameters under working conditions. Two theoretical models, one based on the finite-difference method and one relative to Newton’s law of cooling, are run using a reverse-logic approach. The effect of the different coatings on the convective heat transfer coefficient and the specific heat capacity is evaluated.

### 4.1. Experimental Results

**Figure 5:**
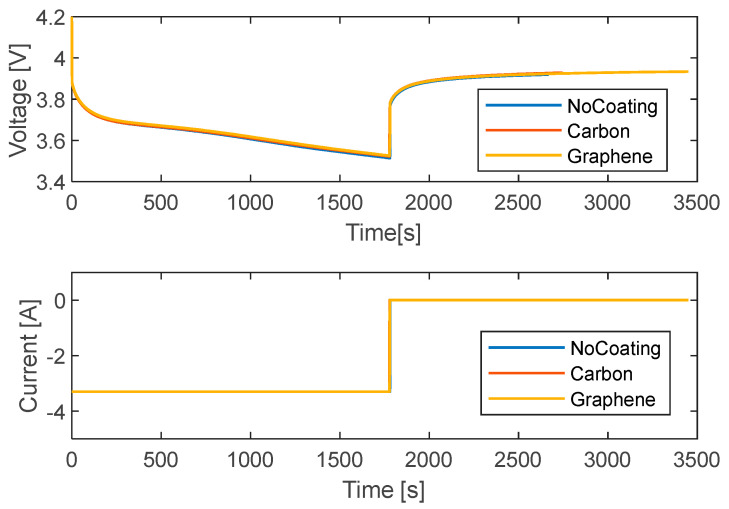
Curves of current and voltage versus time for the discharge tests at 1C with different coatings.

**Figure 6:**
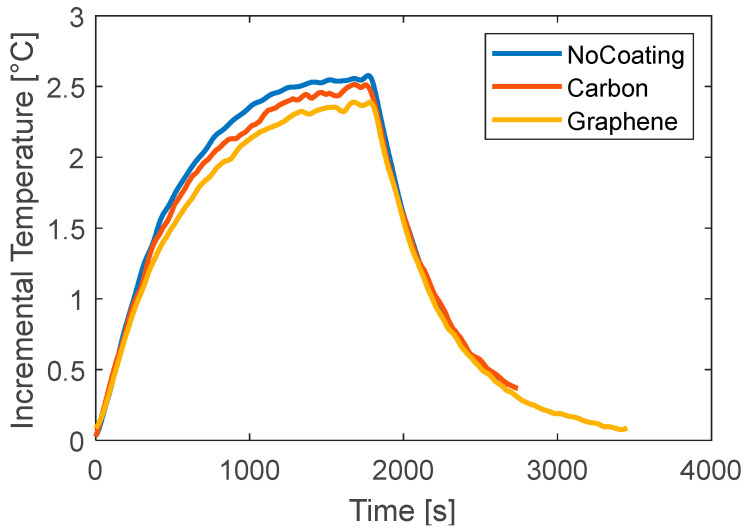
Curves of temperature versus time for different coatings for discharge tests at 1C.

Figure 5 shows the signals of current and voltage for a discharge test at 1C. The initial voltage is 4.2 V for all the curves, as reported in Table 2, a good repeatability among the tests can be appreciated. A rapid voltage drop is experienced when the current is applied because of the battery internal resistance, later it decreases almost linearly over time as it is discharged at a constant current. When the test ends, the voltage is about 3.5 V. At the current cut-off, the voltage signal first rises rapidly up to a certain value depending on both the current and the internal resistance, then it smoothly increases up to a stabilization value. The battery surface temperature for the coated and uncoated cases is reported in Figure 6. The values refer to the net temperature, which is the difference between the battery and ambient temperature.

Starting with the no-coating case, the temperature evolution is characterized by a sharp increment in the first phase and then a plateau. When the current is zero, at 1800 s, the internal heat generation stops and the temperature decreases, smoothly approaching the ambient condition. Using the coatings, carbon and graphene produce slower heating. The maximum increment of temperature is about 2.55 °C, 2.5 °C, and 2.3 °C for the no coating, carbon, and graphene cases, respectively. To better quantify the differences among the curves in Figure 6 during the entire process, the NRMSE is calculated for the carbon and graphene layers in comparison to the no-coating case and reported in Table 3. The NRMSE for graphene, with slightly higher conductivity, is almost three times that of carbon. It can be imagined that the conductive heat transfer toward the coating is more efficient than the convection on the free side of the battery, promoting a higher heat flux toward the coated side than on the optically investigated one. Higher coating conductivity lowers the uncoated side temperature. No significant differences can be appreciated during the cooling phase; where the curves overlap despite the different initial temperatures.

**Figure 7:**
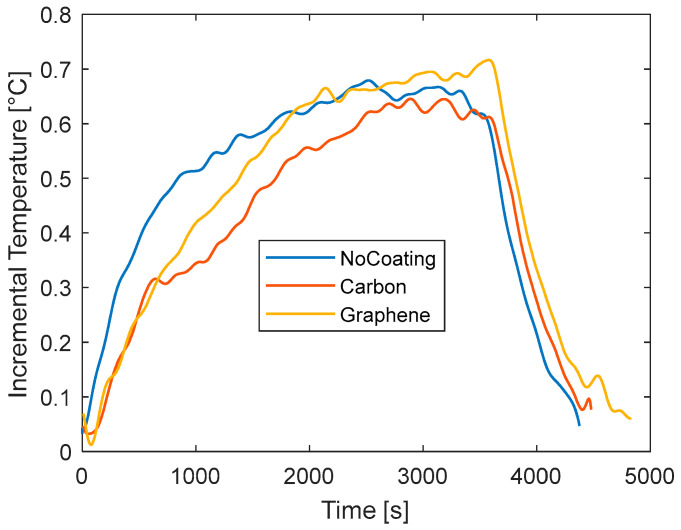
Curves of temperature versus time for different coatings for discharge tests at 0.5C.

Similar considerations can be made for the tests at 0.5C where a lower current is applied. Figure 7 reports the comparison between the three tested cases. The increment of temperature is lower because less heat is generated inside the battery and has a maximum value of about 0.7 °C. In this case, a lower signal-to-noise ratio is obtained; therefore, only the NRMSE (Table 4) is used to make conclusions. The two coatings do not seem to have different behaviors in this low-load case. The NRMSE is comparable. It can be seen that the coatings produce a delay in the increment of temperature at the beginning, converging finally to almost the same temperature as the uncoated case.

**Figure 8:**
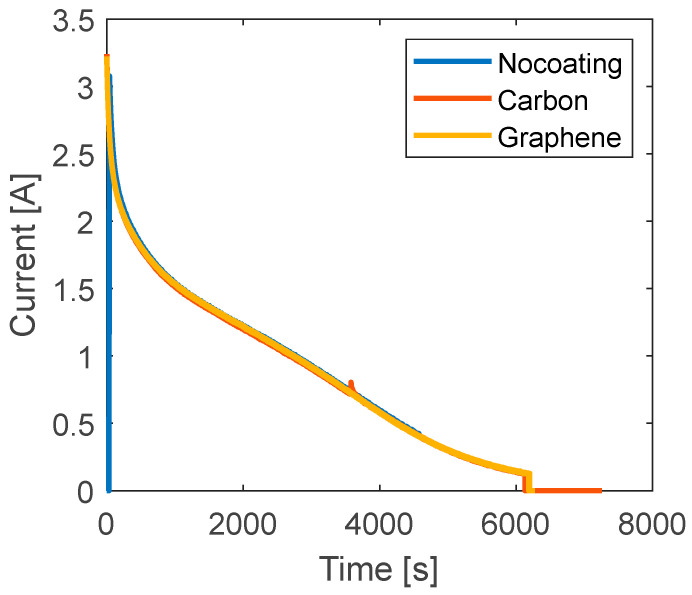
Curves of current versus time for the charge tests with different coating.

**Figure 9:**
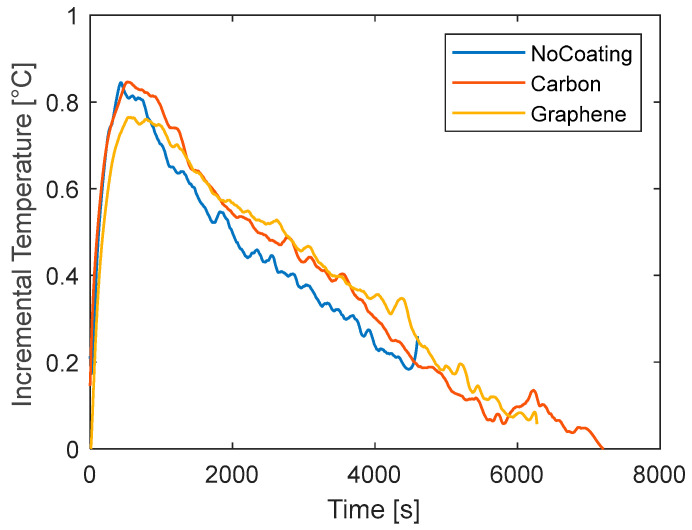
Curves of temperature versus time for different coatings for charge tests.

To this aim, a final set of tests is performed during the charging phase for which the current signals are reported in Figure 8. In these tests, the current is not constant because the CCCV method is applied for the battery charge. It consists in applying a constant current to the battery until the voltage reaches the V_max_ (4.2 V for the battery under investigation) and then maintaining a constant voltage reducing the current. Unlike the previous case, the current decreases over time generating an internal heat that decreases over time. The data of temperature for all the investigated cases under charge are reported in Figure 8 and the calculated NRMSE relative to the no-coating case is reported in Table 5. The curves of temperature variation, reported in the figure differ from those of Figure 7 and Figure 8. A rapid increment of temperature is observed at the beginning due to internal heat accumulation. Then, as the current decreases, the generated heat decreases too. At this point, the heat transferred to the surrounding environment is higher than the internal heat generation, thus resulting in an overall temperature reduction. At the end of the charging phase, the current value is very low and the battery is at ambient temperature. The maximum temperature increment is very low, about 0.8 °C. Most of the test is characterized by a slow cooling down of the battery after the initial rapid heating. From Figure 9, carbon shows the same peak temperature of the no-coating case (between 500 s and 1000 s), while it is lower for graphene. During the cooling phase, the rate of temperature reduction is lower for the coated cases indicating a certain thermal delay induced by the addition of material. The analysis of the NRMSE relative to the no-coating can help to highlight the overall differences during the test. Graphene diverges much from the no-coating case because of the differences both at the peak and during the temperature drop.

Trying to sum up and clarify the obtained results from this analysis at different operating conditions, it seems that the addition of a coating on the battery surface limits the increment of temperature. The effect is most evident for typical current values (1C), whereas for low loads, such as 0.5C, the noise-to-signal ratio alters the results. The layers seem not to affect the cooling phase without internal heat generation. On the contrary, for the charging phase, where the battery cools down even with a very low heat generation, the coatings still provide a thermal delay.

### 4.2. Model Results

The two thermal models previously presented can be used to simulate the temperature of the battery under working conditions. The two main inputs for the determination of the thermal status of the battery are the convective heat transfer coefficient, *h*, and the specific heat capacity, *c_p_*, which are often not available. In addition, their value depends on the temperature and the material, for *c_p_*, and on the temperature gap which is the difference between the initial and maximum temperature during the test, for *h*. Therefore, they can vary from one test to another.

The approach used in the present work for the evaluation of the thermal parameters consists of a reverse calculation using theoretical equations and experimental data of the discharge tests. This approach resembles that used by Zang J. et al. [17] where the numerical results from COMSOL Multiphysics^®^ were compared to the experimental data with an optimization method in modeFRONTIER to determine the thermal parameters that best fit. The present work adopts much simpler models with lower computational costs that still provide a higher accuracy compared to the analysis of Zang X. et al. [44] who also evaluated *h* and *c_p_* using an infrared camera and lumped capacitance method. However, in that case, the battery was heated inside an oven and then cooled down in ambient without applying any current load.

**Figure 10:**
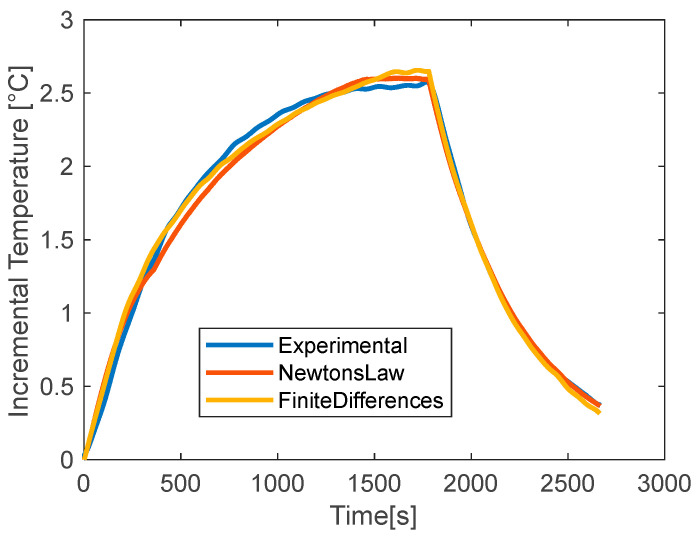
Experimental vs modeled temperature with Newton’s law and finite-differences method for the uncoated battery at discharge rate 1C.

The values of thermal parameters derived in this work are validated by carrying out a comparison between the two models. The good match of the results under the different working conditions has allowed to identify a range of plausibility to consider for future simulations of the battery. First, the finite-difference model is used. A series of curves of temperature is calculated and compared to the experimental data. The one that best fits the measurements is taken and the relative thermal parameters are assumed for that operating condition. To perform the simulation, Equations (3) and (4) are run iteratively for *h* ranging from 20 to 40 W/m^2^K, with a step of 1 W/m^2^K, and *c_p_* from 1000 to 4000 J/kgK, with a step of 100 J/kgK, for a total of 600 modeled curves. Each of them is then compared to the relative experimental curve calculating the NRMSE. The thermal parameters that produce the lowest NRMSE are taken. The second method uses the theoretical correlation of Newton for the cooling and the lumped parameter model, as in [44]. In this case, it is not necessary to perform a minimization of the error because inverting the Equations (7) and (8) as shown in the dedicated section, a single couple of values for *h* and *c_p_* is found, or, better, a couple of values is found for each time step. In general, after some fluctuations and outlier spots corresponding to the initial temperature increment, the values settle around a constant value that is assumed as the final value. This method has, on one side, the advantage of a direct calculation of the optimal thermal parameters starting from the experimental data and ignoring the battery internal specifications. On the other hand, it is limited by the averaging operation just described and by the simplicity of its exponential law that does not always reproduce well the real trend of temperature. Finally, the curves with the lowest NRMSE calculated with the finite difference method and the one re-calculated with the averaged values of *h* and *c_p_* with Newton’s law of cooling for the case at 1C with no-coating are reported in Figure 10, the net temperature is used for the data presentation, that is the difference between the battery and the ambient temperatures. The models have a very good agreement with each other and with the experimental data, both in the heating phase and in the cooling phase. The NRMSE are 0.017 and 0.027 for the curves calculated with the finite-difference method and Newton’s law of cooling, respectively. Using the other coatings, similar results are obtained, and the values of NRMSE for all the investigated conditions are consistent with each other; the values are reported in Table 6. It can be observed that for the two methods, two separated groups of data can be found. The finite-difference method has lower values of NRMSE, from 0.017 to 0.021; while Newton’s law of cooling has higher values, from 0.020 to 0.027. These differences are due to the aforementioned limitations of the second method, which has a fixed exponential temperature profile whatever is the real one.

**Figure 11:**
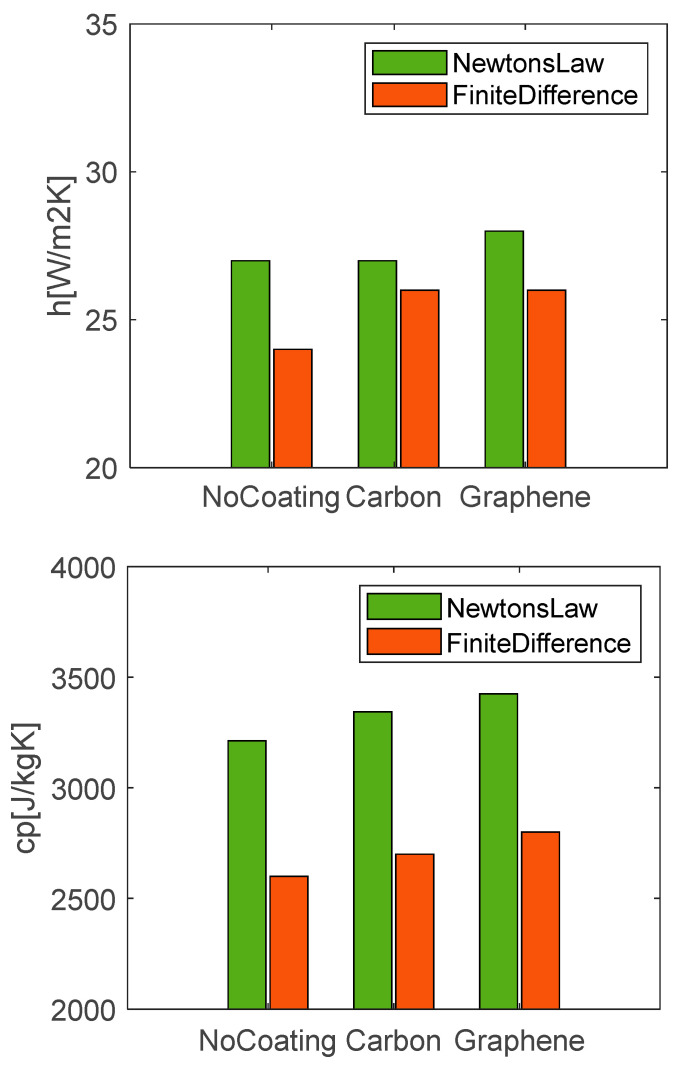
Estimated values of *h* and *c_p_* with Newton’s law and finite-differences method for different coating at discharge rate 1C.

The values of *h* and *c_p_* found for each condition for the discharge case at 1C are reported in the graphs in Figure 11. With regards to the convective heat transfer coefficient, from a general point of view, they are very close from one to the other for the different test cases, being in the range of 24–28 W/m^2^K. These results are expected due to the similar boundary conditions in terms of surrounding fluid properties (air), ambient temperature, and body geometry. Higher values of *h* are obtained moving from the no-coating to the graphene case for which lower temperatures are measured. The estimations made with the two methods are consistent with each other, producing very similar values of *h* for all the conditions. In particular, Newton’s law catches a variation between carbon and graphene; while, the finite difference method estimates a difference between the uncoated and coated samples.

Different considerations can be made for the *c_p_.* First, an increasing trend moving from the no-coating case to the carbon and then graphene is observed for both the used methods. But the thermal parameter has lower values, from 2600 to 2800 J/kgK, for the finite-difference method, and higher ones, from 3200 to 3400 J/kgK, for Newton’s law of cooling; whereas, the value of 2600 J/kgK obtained for the no-coating case with the first method is very close to the average specific heat capacity of 2500 J/kgK found by Wang et al. [43] for a Li-ion battery using the weighted average method, calculated according to the mass fraction and specific heat capacity of each part of the cell. The agreement of the estimated parameters to the literature and the lower measured NRMSE support the reliability of the finite-difference method for the estimation of the thermal parameters in place of the simplest but less accurate Newton’s law of cooling.

**Figure 12:**
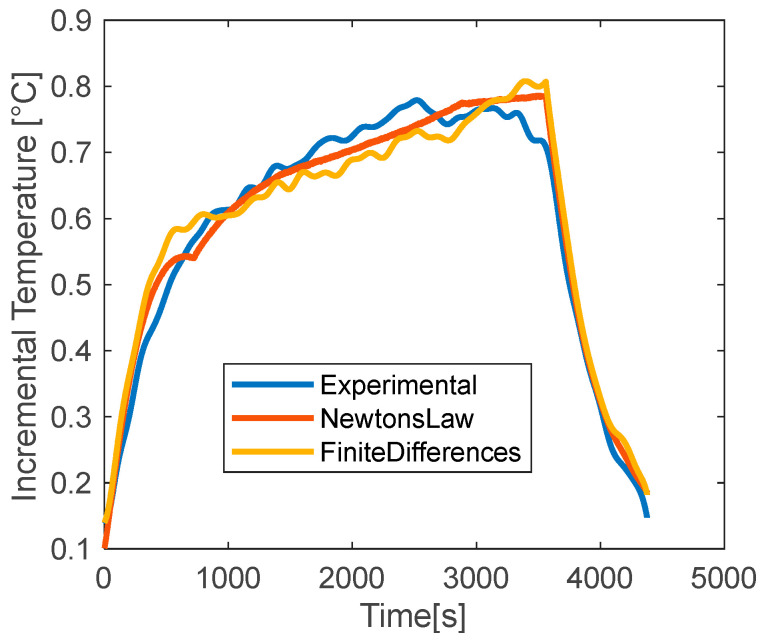
Experimental vs modeled temperature with Newton’s law and finite-differences method for the uncoated battery at discharge rate 0.5C.

**Figure 13:**
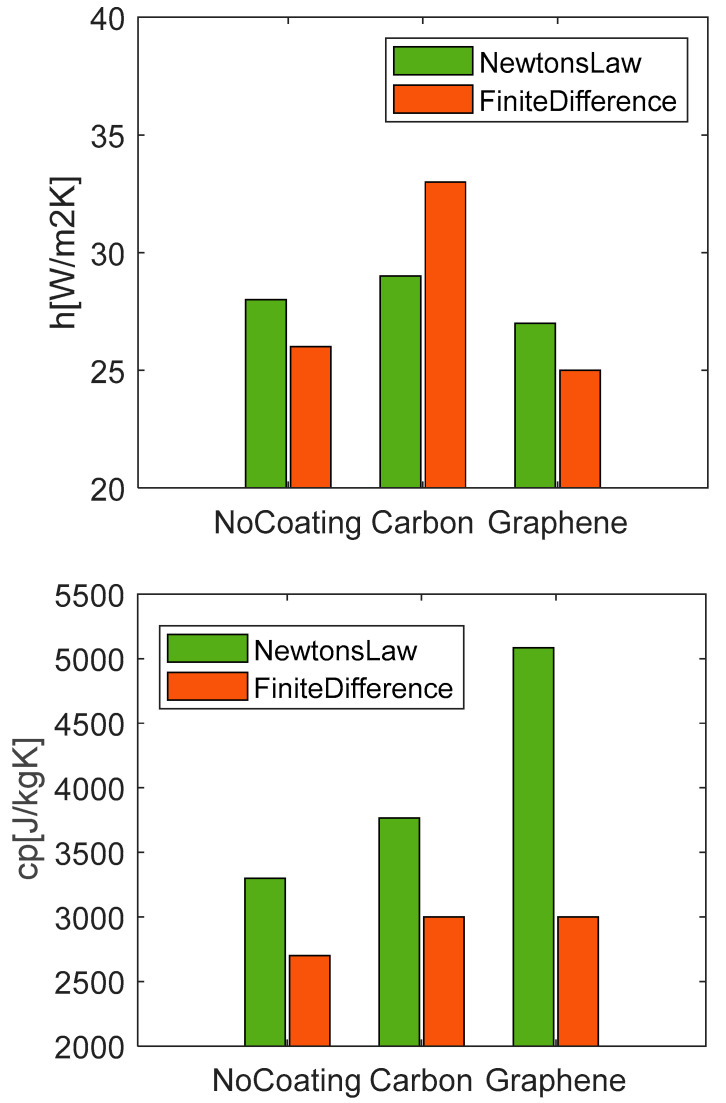
Estimated values of *h* and *c_p_* with Newton’s law and finite-differences method for different coating at discharge rate 0.5C.

To further analyze the performances of the two methods, evaluations are also run with 0.5C discharge current. Figure 12 shows a good agreement between the experimental and the model curves, but with less accuracy than in the previous case. It can be seen that the experimental data have some oscillations that are well reproduced by the finite-difference method whereas they are approximated by a straight line by Newton’s correlation. A good match is obtained for both during the cooling phase. Table 7 shows the NRMSE values for the two applied methods. Both models have higher errors than the previous case (Table 6) for all tests. And the data of carbon has an NRMSE value one order of magnitude higher than the others. The low accuracy of this test case is likely due to the small variation of temperature, of about 0.8 °C. The uncertainty of the thermal sensor has a higher impact on the absolute value of the measure, which often leads to data fluctuations. As a consequence, the evaluation of the thermal parameters is less accurate. Figure 13 compares the evaluated values of *h* at 0.5C of discharge current. Differently from the previous case, neither of the two methods presents a trend passing from one coating to another. Moreover, the data span, from 25 to 34 W/m^2^K, is almost doubled for the finite-difference method with the average value being close to the previous case at 1C. On the other side, the analysis of the *c_p_* provides interesting insights because both the used methods reveal a trend from the no-coating to the graphene case; again, a positive offset is obtained for the results of Newton’s correlation. In general, it seems that low values of *c_p_* are obtained for the low conductivity coatings.

The values estimated for the different current loads, coatings, and methods used show an overall good agreement, indicating that the presented approaches are a viable and fast solution for the evaluation of the unknown thermal parameters of a battery starting from temperature measurements during simple working conditions and non-stationary cases. Moreover, this methodology seems to be sensitive to the coating providing small differences according to the material conductivity. On the other hand, some limitations are visible for Newton’s correlation whilst the finite-difference method works better in different conditions.

## 5. Conclusions

In a scenario where the thermal management of battery-based systems has become a crucial aspect to consider from the design phase to the application, this work expands the outcomes of previous investigations on the thermal status of energy storage systems [45,46,47] and contributes to the development of methodologies to find the battery thermal parameters, such as the convective heat transfer coefficient and the specific heat capacity. Temperature measurements during discharge tests with two working currents are performed by coating the battery with different materials varying the thermal conductivity. Then, two theoretical models are used with a reverse-logic approach for the estimation of the thermal parameters of the battery using the experimental temperature data.

The battery temperature increases during the discharge process without reaching a stable phase for both the current values in the test under investigation. The lack of a stable part, where the temperature derivative is null, prevents from using the thermal balance for a rapid evaluation of the convective heat transfer coefficient. For the two current values, higher currents produce higher temperature gaps, the increment is about 2.5 °C and 0.8 °C, at 1C and 0.5C current ratings, respectively. Moreover, a lower signal-to-noise ratio is obtained for the low current test. Concerning the coating materials, the lowest temperature is obtained with graphene which is slightly more conductive than carbon.

To evaluate the unknown thermal parameters of the battery under non-stationary conditions, the two theoretical correlations are applied and compared. The finite-difference method is used iteratively by minimizing the NRMSE between the experimental and model curves, while Newton’s law of cooling is mathematically inverted to calculate the time-averaged values. The convective heat transfer coefficient and the specific heat transfer increase with the thermal conductivity of the coatings. In general, when an additional layer is applied to the battery, the thermal parameters increase. For the specific heat capacity, this can be due to the addition of mass, while for the convective heat transfer coefficient, the correlation is less direct due to the ability to dissipate the same heat flux with a lower delta of temperature between the battery and the ambient. With regards to the two proposed methods, they agree and are sensitive to the coating material even with variations of low extent, revealing to be suitable for the evaluation of the unknown thermal parameters of a battery starting from temperature data. More reliable results are obtained for the discharge at 1C where the lowest difference between the two methods is found for *h*. Moreover, in the case of signal fluctuations or low signal-to-noise ratio, the finite-difference method is more appropriate as it can reproduce the oscillations of the experimental curve.

Besides the investigation and discussion of the battery temperature, this work provides experimentally derived values of the two main thermal parameters of a common-type Li-ion battery that can be used for modeling. Additionally, the comparison among the coating conductivities can be scaled to different applications or configurations. Also, the proposed methods for the estimation of the parameters are largely applicable to various applications. Future plans will concern the validation of the thermal parameters found in this work to non-stationary conditions for the same experimental configuration; the assessment of the best method with different battery types; and the experimental set-up and testing of battery pack configurations with on-purpose designed heat dissipation systems based on the carbon technology.

## Figures and Tables

**Table 1:** Values of conductivity.

Coatings	Carbon	Graphene
Conductivity [W/mK]	0.67	0.86
Battery conductivity measured across the transverse profile = 1.25 W/mK		

**Table 2:** Test plan specifications.

Test	Initial-Final SOC	Initial Voltage	Current Rating	Charge/Discharge Time	Resting Time
1	100–50%	4.2 V	0.5C	3600 s	1800 s
2	100–50%	4.2 V	1C	1800 s	1800 s
3	50–100%	3.9 V	CCCV method	Variable	0 s

**Table 3:** NRMSE between the curve of temperature for the uncoated and coated battery at discharge rate 1C.

	NRMSE
Discharge 1C carbon	0.03
Discharge 1C graphene	0.10

**Table 4:** NRMSE between the curve of temperature for the uncoated and coated battery at discharge rate 0.5C.

	NRMSE
Discharge 0.5C carbon	0.19
Discharge 0.5C graphene	0.16

**Table 5:** NRMSE between the curve of temperature for the uncoated and coated battery during charge mode.

	NRMSE
Charge-carbon	0.16
Charge-graphene	0.27

**Table 6:** NRMSE between experimental and modeled curves of temperature at discharge rate 1C.

NRMSE	Model Based on the Finite-Difference Method	Model Based on Newton’s Cooling Law
Discharge 1C no-coating	0.017	0.027
Discharge 1C carbon	0.018	0.025
Discharge 1C graphene	0.021	0.020

**Table 7:** NRMSE between experimental and modeled curves of temperature at discharge rate 0.5C.

NRMSE	Model Based on the Finite-Difference Method	Model Based on Newton’s Cooling Law
Discharge 0.5C no-coating	0.027	0.043
Discharge 0.5C carbon	0.26	0.13
Discharge 0.5C graphene	0.041	0.071
